# What is the relationship between hospital management practices and quality of care? A systematic review of the global evidence

**DOI:** 10.1093/heapol/czae112

**Published:** 2024-11-22

**Authors:** Charlotte Ward, Elias Rejoice Maynard Phiri, Catherine Goodman, Alinane Linda Nyondo-Mipando, Monica Malata, Wanangwa Chimwaza Manda, Victor Mwapasa, Timothy Powell-Jackson

**Affiliations:** Department of Global Health and Development, London School of Hygiene and Tropical Medicine, 15-17 Tavistock Place, London, WC1H 9SH, United Kingdom; Health Systems Department, Kamuzu University of Health Sciences, Blantyre, Malawi; Department of Global Health and Development, London School of Hygiene and Tropical Medicine, 15-17 Tavistock Place, London, WC1H 9SH, United Kingdom; Health Systems Department, Kamuzu University of Health Sciences, Blantyre, Malawi; Health Systems Department, Kamuzu University of Health Sciences, Blantyre, Malawi; Health Systems Department, Kamuzu University of Health Sciences, Blantyre, Malawi; Health Systems Department, Kamuzu University of Health Sciences, Blantyre, Malawi; Department of Global Health and Development, London School of Hygiene and Tropical Medicine, 15-17 Tavistock Place, London, WC1H 9SH, United Kingdom

**Keywords:** management practices, quality of care, systematic review, health systems

## Abstract

There is a widely held view that good management improves organizational performance. However, hospitals are complex organizations, and the relationship between management practices and health service delivery is not straightforward. We conducted a global, systematic literature review of the quantitative evidence on the link between the adoption of management practices and quality of care in hospitals. We searched in PubMed, EMBASE, EconLit, Global Health, and Web of Science on 16 October 2024, without language or country restrictions. We included empirical studies from 1 January 2000 onwards, examining the quantitative association between hospital management practices and quality of care. Outcomes included structural quality (availability of resources such as drugs and equipment), clinical quality (adherence to guidelines), health outcomes, and patient satisfaction or experience with care. In every study, each tested association was categorized as significantly positive (at the 5% level), null, or significantly negative. The study was registered with PROSPERO (CRD42022301462). Of 11 731 articles, 25 studies met the inclusion criteria and had an acceptable risk of bias. Studies were equally distributed between high-income and low- and middle-income countries, with 22 cross-sectional and three intervention studies. Of 111 associations, 55 (49.5%) were significantly positive, one (1%) was significantly negative, and 55 (49.5%) were null. Among the associations tested, the majority were significantly positive for structural quality (79%), clinical quality (60%), and health outcomes (57%), while most associations between hospital management and patient satisfaction (80%) were null. The findings are mixed, with a similar proportion of positive and null associations between management practices and quality of care across studies. The evidence is limited by the risk of bias introduced by nonrandomized study designs. Evidence of positive associations in some settings warrants further investigation of the association through intervention studies or natural experiments. This could leverage methodological developments in quantitatively measuring management, highlighted by this review.

Key messages

- There is mixed global evidence on the relationship between management practices and quality of care in hospitals.
- The current evidence is limited due to the risk of bias introduced by nonrandomized study designs.
- Evidence of positive associations in some settings warrants further investigation of the association through intervention studies or natural experiments.
- To provide methodological learning, we supplemented traditional manual screening methods with active learning software and documented how studies have quantitatively measured management practices.

## Introduction

Ensuring high quality of care is widely recognized as essential if countries are to make meaningful progress towards universal health coverage (Kruk et al. 2018). Estimates suggest that poor quality of care accounts for between 5.7 and 8.4 million deaths each year in low- and middle-income countries (LMICs) (World Health Organization 2020). During the Millennium Development Goals era (2000–15), global and national policy was focused primarily on improving coverage of essential health services. However, over the last decade, quality of care has risen up the policy agenda in all income settings (World Health Organization 2015, Kruk et al. 2018).

To identify potential interventions and policies to improve quality of care, a strong evidence base is required on its determinants. While an enormous number of controlled studies have been conducted on the effect of health technologies (Darmstadt et al. 2005), medical training, and clinical audits (Rowe et al. 2018) on quality of care and patient outcomes, much less research has assessed the broader organization-level factors that potentially shape quality of care (Sheikh et al. 2011, Kruk et al. 2018). One such factor is hospital management, which has long been recognized by the press and policymakers as important (Murray and Frenk 2000), but has received limited attention from empirical researchers (Rumbold et al. 2015).

Management has been variously described as ‘the control, monitoring or organization of people, processes and systems in order to achieve specific goals’ (The Health Foundation 2022) or ‘continuously developing the potential of an organization to transform human and financial resources and other inputs into improved services and better health’ (Vriesendorp et al. 2010). It is clear that management is multidimensional, implying that there is no single indicator of management. This presents a significant measurement challenge. Moreover, what amounts to ‘good management’ can often include both objective and more subjective elements, reflecting, e.g., the perceived quality of managerial actions, and heterogenous preferences for different management styles.

To address these challenges, researchers have increasingly conceptualized management as the adoption of management practices (Bloom and Van Reenen 2007). This implies a focus on processes and systems that are implemented in an organization. Some management practices lack consensus by experts or a strong evidence base to be evaluated as good or bad (Scur et al. 2021). However, other practices—from promoting staff who are performing well to continuous tracking of key performance indicators—are widely accepted as being beneficial to the performance of any organization, and quantitative tools tend to focus on these practices to measure management. Management practices are often organized into domains, with common examples including human resource management, performance monitoring, target setting, financial management, and (lean) operations (West et al. 2006, Tsai et al. 2015, Fetene et al. 2019).

Whether the adoption of certain management practices is likely to improve quality of care in hospitals is far from certain. On the one hand, there is strong quantitative evidence from manufacturing firms that management practices matter for performance, and there are similar findings for other sectors such as retail and education (Bloom et al. 2012, 2015a, Mccormack et al. 2014, Scur et al. 2021). Logically, one would expect practice in domains such as human resource management to be just as important for a hospital as for any other organization. Moreover, high-profile inquiries highlight stark examples of how incompetent hospital management has led to catastrophic failures in patient care (Francis 2013). On the other hand, hospitals differ from other organizations in ways that could weaken the link between management practices and performance. First, there is not always a clear separation between managerial and clinical roles in hospitals (Nzinga et al. 2019), and it is conceivable that excessive management tasks reduce clinicians’ time for clinical care. Secondly, hospitals often face competing demands to both deliver high-quality care and be financially sustainable. If management practices are orientated more to the latter, service delivery and quality of care may suffer (Mckay and Deily 2008, Stargardt et al. 2014). Thirdly, the nature of incentives faced by managers of public or not-for-profit hospitals (Bénabou and Tirole 2006), as well as a lack of robust, routine measurement of quality of care, may lead managers to focus on bureaucratic tasks with little or no benefit to patients (Veronesi et al. 2019).

To the best of our knowledge, no systematic review has synthesized the quantitative evidence on the relationship between hospital management practices and quality of care. Earlier reviews have explored elements of this question but were all conducted over a decade ago, covering a period largely before the development of tools to measure hospital management practices (Mazzocato et al. 2010, Lega et al. 2013, Parand et al. 2014). The paper most closely related to ours was a narrative review, including both theoretical and empirical literature (both quantitative and qualitative), and covering a broad range of topics on the role of management in healthcare systems (Lega et al. 2013).

This systematic review aimed to summarize the quantitative evidence on the relationship between hospital management practices and various dimensions of quality of care, drawing on evidence from all income settings. We also describe the range of tools used in studies to measure management practices.

## Materials and methods

### Protocol and registration

This study is registered with the PROSPERO international prospective register of systematic reviews (registration number CRD42022301462). We developed a protocol to guide the conduct of the review and report findings according to Preferred Reporting Items for Systematic reviews and Meta-Analyses (PRISMA) guidelines (Page et al. 2021).

### Eligibility criteria

Studies were eligible for inclusion if they were (i) empirical research studies (excluding guidelines, opinion pieces, and reviews); (ii) studying the association between management practices as an exposure and quality of care as an outcome; (iii) conducted fully or partially in the hospital setting; (iv) had an abstract and full-text available; and (v) were published from 2000 onwards. Drawing on Donabedian’s quality of care framework (Donabedian 1988), we included studies reporting outcomes for structural quality (availability of inputs), process quality (clinical care given to patients), and patient outcomes (patient health, patient satisfaction, and experience of care).

Quantitative studies of any design were considered for inclusion. We included studies that examined the association between management practices and quality of care through either an observational study or an evaluation of a management intervention. Any intervention study design was included, provided that the study demonstrated the association between the management intervention and both the adoption of management practices and a quality of care outcome.

### Information sources and search strategy

Our search included five databases: PubMed, EMBASE, Global Health, EconLit, and Web of Science Core Collection. A Boolean search strategy was developed for PubMed incorporating MeSH terms, truncated search terms, and synonyms and adapted for the other four databases (Tables A1–A4 in the Supplementary material). The search strategy combined three concepts: management (e.g. ‘hospital management’, ‘health care management’, ‘management score’, and ‘management performance’), quality of care (e.g. ‘hospital performance’, ‘clinical standard’, and ‘quality of health care’), and hospital setting (e.g. ‘hospital’, ‘department’, and ‘ward’). Databases were searched on 1 August 2022 and again on 16 October 2024. We supplemented the search by scanning the reference lists of the studies included in the review using Scopus. We removed duplicate references using Rayyan software.

### Study selection

We combined the traditional manual screening methods with active learning software called ASReview LAB Version 1.0 (Van De Schoot et al. 2021). This allowed us to be expansive in our search strategy and more efficient in our screening of titles and abstracts. ASReview is an open-source platform that uses machine learning to prioritize the next article to be screened by the reviewer based on previous selections from the set of records obtained from the systematic search. ASReview was useful for this type of review because we were using broad search terms that are applicable to several research areas, and therefore the deduplicated search results contained a very large number of records. While such software can be used to reduce screening to the top *x*% of articles prioritized by such software (Cohen et al. 2006, Van De Schoot et al. 2021), we screened all papers to minimize the risk of missing relevant papers. We used ASReview to prioritize the top 10% for independent review by two reviewers, while the titles and abstracts of the remaining 90% were manually screened using Rayyan software by one reviewer. Relevant articles were retrieved for full-text review by two independent reviewers. Disagreements were resolved through group consensus, and reasons for exclusion were fully documented.

### Data items and extraction

A structured template was used to extract characteristics of the studies, including authors, year of publication, study design, country, country income category, sector of providers studied, study setting (hospital, hospital department, health centre, clinic, and dispensary), type of management measure, content of the management measure, name of survey tool, number of indicators collected, data collection method, type of responses, source of information, format of responses, management measure score, measure of spread, intervention description, outcome, sample size, statistical analysis, details of adjustment for confounding or clustering, nature of coefficient, and coefficient value and measure of spread. Data from relevant studies were extracted by one reviewer and independently checked by a second reviewer.

Some studies performed multiple analyses. We extracted the results of each analysis if a study examined the association between management practices and multiple quality of care outcomes. For studies that defined management in multiple ways, we extracted results for the primary measure of management (or all results if no primary measure was specified). In studies assessing the association between varying levels of management practice adoption and quality of care, we extracted results by comparing the reference category with the level showing the greatest contrast. Finally, for studies that performed multiple adjustments for the same exposure and outcome, we extracted results from the authors’ preferred regression model. We did not extract results for subgroup analyses or outcomes that were not a measure of quality of care.

### Risk of bias assessment

We assessed for bias based on domains from the ROBINS-I tool (Sterne et al. 2016): confounding, participant selection, classification of exposure (management practices), deviation from intervention, missing data, outcome measure (quality of care), and selection of reported result (Table A5 in the Supplementary material). Within each domain, studies were scored as having low, moderate, serious, or critical risk of bias, or no information. For studies that conducted a primary analysis comprising more than one type of quality of care outcome, we performed the risk of bias assessment for each analysis and, where this made a difference to the scoring, reported the risk of bias results separately. Overall bias was based on the worst scoring domain and studies with ‘critical’ risk of bias were excluded from the analysis. This means that the study was too problematic in this domain to provide any useful evidence on the association of interest.

### Summary measures, data synthesis, and analysis

We analysed the findings by the type of quality of care measure (Donabedian 1988). For each analysis, we categorized the association between management practices and quality of care as significantly positive, significantly negative, or null. A positive association meant that better management practices had a statistically significant association with higher quality of care. We used a 5% level to determine statistical significance. For studies that used multiple indicators or domains of management practices without specifying a primary measure, we considered each set of associations as an analysis and categorized the result as positive if at least one association was positive. We did not account for multiple hypothesis testing. We could not do a meta-analysis due to heterogeneity in the way that studies define and measure management practices and quality of care.

We report the number and proportion of significantly positive, negative, or null associations overall and by country income setting, outcome, and risk of bias assessment level. At the study level, we categorized the proportion of associations that were positive as ‘all positive’, ‘majority positive’ (≥50%), ‘minority positive’ (<50%), and ‘all null’.

## Results

### Study selection

**Figure 1.**
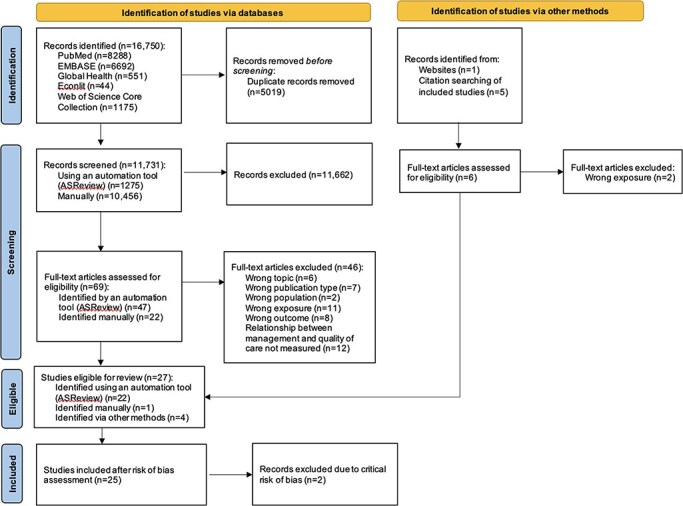
PRISMA 2020 flow diagram.

Figure 1 provides a summary of the study selection process according to PRISMA guidelines (Moher et al. 2009). A total of 11 731 unique records were identified in database searches and a further six through websites and citation searching. Of these, 75 were accepted for full-text review, of which 27 were eligible for inclusion prior to the risk of bias assessment. Among eligible studies identified through the first database search (date duration 1 January 2000–1 August 2022), all were identified using the automation tool ASReview, meaning that the subsequent manual search identified no additional studies. Records were screened manually during the second database search (date duration 2 August 2022–16 October 2024), and one additional study was identified. Four studies (Pollack and Koch 2003, West et al. 2006, King et al. 2021, Powell-Jackson et al. 2024) were sourced from websites or searching citations of the eligible studies.

### Risk of bias

Among the 27 studies eligible for inclusion, 17 had a moderate risk of bias, eight had a serious risk of bias, and two had a critical risk of bias and were excluded, leaving 25 studies for inclusion in the evidence synthesis (Table A6 in the Supplementary material). Except for one study, all had at least a moderate risk of bias due to potential confounding introduced through a cross-sectional study design or a before and after intervention study. A serious risk of bias was most frequently introduced through the way management was defined and measured. For example, several studies (Asaria et al. 2021, Byabagambi et al. 2017, Mwencha et al. 2017, Salehnejad et al. 2022, Thatte and Choi 2015) measured management practices using a small number of items without a clear rationale for selecting them or relied on subjective measures of management based on staff’s opinion of their managers.

### Study settings and characteristics

A descriptive summary of the studies is presented in Table 1 and data extracted for each individual study is presented in Table A7 of the Supplementary material. The 25 studies included were equally distributed between high-income countries and LMICs. All studies investigated the management–quality association using a cross-sectional design, with the exception of two before and after intervention studies (Byabagambi et al. 2017, Mwencha et al. 2017) and one randomized controlled trial (King et al. 2021). The interventions in these studies sought to address supply chain management (Mwencha et al. 2017), pharmaceutical and human resource management (Byabagambi et al. 2017), and multiple domains of management (King et al. 2021). Fourteen studies reported evidence only from hospitals and the remaining 11 included both hospitals and other types of facilities including health centres, clinics, and dispensaries. Nineteen studies summarized the measure of management with a summary index and six measured individual items of management. To measure management practices, five studies used the World Management Survey and eight adapted it. Others used data from surveys about staff’s perception of management (Asaria et al. 2021, Salehnejad et al. 2022) or grouped certain characteristics using cluster analysis to create different managerial models (Fanelli et al. 2020). Some studies focused on a specific area of management such as human resources (Thatte and Choi 2014, West et al. 2002, 2006), supply chain management (Mwencha et al. 2017), or pharmaceutical management practices (Byabagambi et al. 2017). More information about what was included in the management measure(s) can be found in Table 2.

**Table 1.** Characteristics of the studies included in evidence synthesis

	Number of studies	%
Income setting		
High-income	12	48.0
Low- and middle-income	13	52.0
Study design		
Cross-sectional association	22	88.0
Before and after intervention	2	8.0
Randomized controlled trial	1	4.0
Health facility type		
Hospital only	14	56.0
Hospitals and other types of facilities	11	44.0
Sector		
Private	3	12.0
Public	6	24.0
Private and public	13	52.0
Not stated	3	12.0
Management measure		
World Management Survey	5	20.0
Adapted from the World Management Survey	8	32.0
Other	12	48.0
Proportion of positive management–quality associations (at the 5% level)		
All associations	8	32.0
Majority (≥50%) of associations	8	32.0
Minority (<50%) of associations	3	12.0
No associations	6	24.0

**Table 2.** Description of the management measures used in eligible studies

Author (year)	Name of survey tool	Type of management measure (index or multiple items of management)	What was included in the management measure?
Acharya et al. (2022)	Other	Multiple items of management	Monthly management meetings, external supervision in last 4 months, routine quality assurance activities, and system for collecting opinion and review
Adhikari (2024)	Other	Multiple items of management	Quality assurance performed, external supervision, system to take client opinion, and frequency of monthly health facility meeting
Adler-Milstein et al. (2014)	World Management Survey	Index	Operations, performance, targets, and talent
Asaria et al. (2021)	Other	Index	Perceptions of managerial quality based on 11 questions from a National Health Service staff survey. Three out of 11 questions are focused on management practices of senior managers (decisions, feedback, and communication)
Bloom et al. (2015b)	World Management Survey	Index	Operations and monitoring, targets, incentives management
Bloom et al. (2020)	World Management Survey	Index	Operation, monitoring, targets, and human resources
Byabagambi et al. (2017)	Other	Multiple items of management	Pharmaceutical management practices measured, e.g. standard operating procedures available, stock cards available, job descriptions documented, and system to monitor stock count
Fanelli et al. (2020)	Other	Index	Informed by a framework, the authors define five areas within which managers make choices: performance and results review; benchmarking; leadership; clinical guidelines, protocols, and procedure; and staff satisfaction. These five areas are used to construct their management measure
Groene et al. (2015)	Other	Index	Quality policies, procedures, and activities (such as quality monitoring, infection control, and complaints handling)
Kim et al. (2022)	Adapted from the World Management Survey	Index	Target setting, operations, human resources, monitoring and evaluation, and community engagement
King et al. (2021)	Other	Index	Governance and management, human resource management, patient and family rights and access to care, management of information, risk management, primary healthcare services, in-patient care, surgery and anaesthesia services, laboratory services, diagnostic imaging services, medication management, facility management services, and support services
Macarayan et al. (2019)	Adapted from World Management Survey	Index	Target setting, operations, human resources, monitoring, and community engagement
Mcconnell et al. (2013)	World Management Survey	Index	Operation management, performance, targets, and talent
Mwencha et al. (2017)	Other	Index	Quantification, storage, transportation, inventory management, logistics data management, monitoring and control, design, and planning
Plough et al. (2017)	Adapted from the World Management Survey	Index	Team collaboration, communication and coordination, obstetrician availability, obstetrician shared patient responsibility, quality improvement engagement, labour floor efficiency, commitment to vaginal delivery, dynamic resource management, bottlenecks, flexible physical capacity, planned case scheduling, conflict management, patient assignment, and flexible nurse staffing
Powell-Jackson et al. (2024)	Adapted from the World Management Survey	Index	Operations, performance monitoring and targets, human resource management, and financial management
Salas-Ortiz et al. (2019)	Adapted from the World Management Survey	Index	Performance-based funding, sanctions, external supervision, community participation, national-level governance, and municipal-level governance
Salehnejad et al. (2022)	Other	Multiple items of management	Decentralization of decision-making, effective communication, acting on staff feedback, acting on ideas for improving services, flexible working practices, workplace pressure, incident reporting, job design, appraisal, and supervisor and team quality
Thatte and Choi (2015)	Other	Multiple items of management	Provider trained, provider received supervision, and provider had a written job description
Tsai et al. (2015)	World Management Survey	Index	Operations, monitoring, targets, and human resources
Wang et al. (2022)	Adapted from World Management Survey	Index	Standardized operations, performance monitoring, target setting, and talent management
West et al. (2002)	Other	Multiple items of management	Human resource management practices and policies
West et al. (2006)	Other	Index	Training, performance management, participation, decentralization, involvement, use of teams, employment security, and investor in people status
Yoo et al. (2019)	Adapted from the World Management Survey	Index	Operations, performance, targets, and talent
Zhu et al. (2021)	Adapted from the World Management Survey	Index	Target, performance, efficacy, and talent

Structural quality centred around whether the health care provider had drugs, equipment, staff, and guidelines available and was measured through a health facility survey (Macarayan et al. 2019, Acharya et al. 2022, Kim et al. 2022, Adhikari et al. 2024) or a supply chain routine information system (Mwencha et al. 2017).

Clinical quality was concerned with the providers’ compliance with care guidelines, such as infection prevention and control protocols, case management guidelines, or management of acute myocardial infarction (Mcconnell et al. 2013, Byabagambi et al. 2017, Salas-Ortiz et al. 2019, King et al. 2021, Powell-Jackson et al. 2024). A notable exception (Byabagambi et al. 2017) also measured the patients’ adherence to treatment and whether they could explain their treatment correctly. The authors used standardized patients (King et al. 2021, Powell-Jackson et al. 2024), direct observation (King et al. 2021, Thatte and Choi 2015, Powell-Jackson et al. 2024), clinical records (Mcconnell et al. 2013, Byabagambi et al. 2017), and clinical vignettes (Salas-Ortiz et al. 2019) to measure clinical quality.

Health outcomes comprised general mortality indicators (West et al. 2006, Asaria et al. 2021, Salehnejad et al. 2022), those measuring mortality from acute myocardial infarction (Mcconnell et al. 2013, Adler-Milstein et al. 2014, Bloom et al. 2015b, 2020, Yoo et al. 2019), or those measuring mortality or morbidity from other conditions of interest (West et al. 2002, Byabagambi et al. 2017, Plough et al. 2017, Fanelli et al. 2020). Health outcomes were measured through routinely collected data from secondary sources (West et al. 2006, Mcconnell et al. 2013, Adler-Milstein et al. 2014, Bloom et al. 2015b, 2020, Plough et al. 2017, Yoo et al. 2019, Asaria et al. 2021, Salehnejad et al. 2022) such as a hospital information system or a national survey or primary data sources such as record review or survey (West et al. 2002, Byabagambi et al. 2017, Fanelli et al. 2020).

Patient-reported outcomes were closely linked to the World Health Organization’s conceptualization of responsiveness (Darby et al. 2003) and focused on respect, trust in providers, clear communication, information provision, perceived involvement in care, quality of service, and likelihood of recommending or returning to the hospital. Studies measuring these outcomes asked patients to rate their satisfaction or experience with care using a Likert scale (Macarayan et al. 2019, Kim et al. 2022, Wang et al. 2022) or captured information with other closed-ended questions (Groene et al. 2015). With one exception (Byabagambi et al. 2017), all studies controlled for confounding in their analysis. Potential confounders included facility variables (e.g. type of facility, rurality, teaching status, budget, and number of beds), patient characteristics (age, gender, and sociodemographic variables), interviewer characteristics, and interviewee characteristics (e.g. department, position, and tenure).

### Association between management and quality

In eight (32%) studies, all associations were significantly positive, and in six (24%) studies all were null (Table 1). Of 111 associations, 55 (49.5%) were significantly positive, one (1%) was significantly negative, and 55 (49.5%) were null (Table A7 in the Supplementary material).

**Figure 2.**
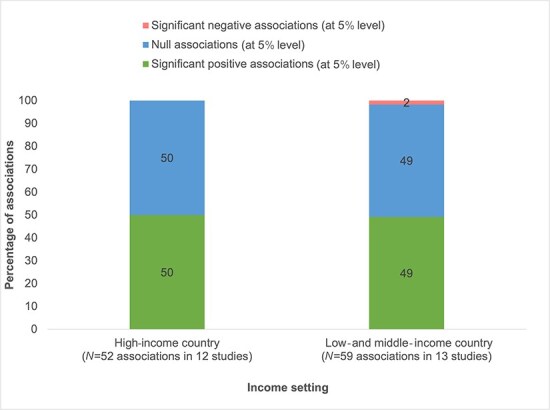
Management–quality associations, by income setting.

**Figure 3.**
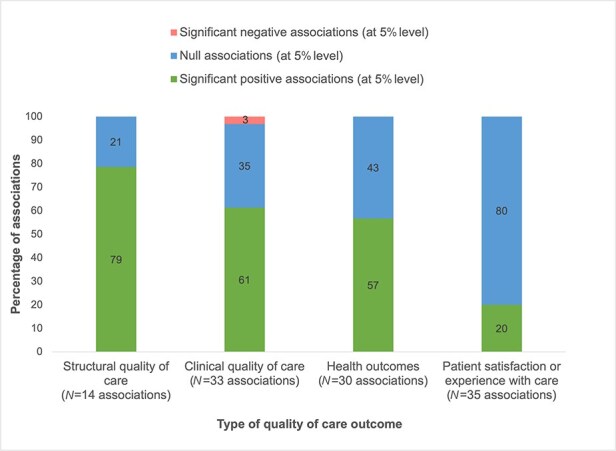
Management–quality associations, by type of quality of care outcome.

**Figure 4.**
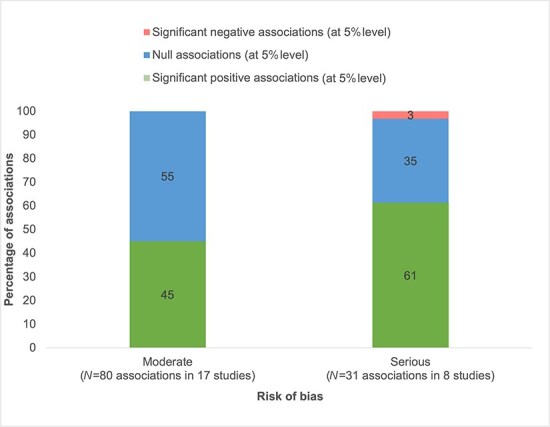
Management–quality associations, by risk of bias categorization.

Figure 2 shows results on the association between management practices and quality of care by income setting. There is little difference between the proportion of significant positive and null associations between studies set in high-income countries versus LMICs. Figure 3 presents results on the management–quality association for different types of quality of care outcomes. Of the 14 (79%) associations between management and structural quality of care, 11 were positive. Of the 31 (61%) associations between management and clinical quality, 19 were positive, and of 30 (57%) associations between management and health outcomes, 17 were positive. For outcomes measured through patient satisfaction or experience with care, seven out of 35 (20%) associations are positive at the 5% level. Across all quality of care outcomes, for studies categorized with a moderate risk of bias, 36 out of 80 (45%) associations are positive compared to those categorized with a serious risk of bias where 19 out of 31 (61%) associations are positive (Fig. 4).

**Figure 5.**
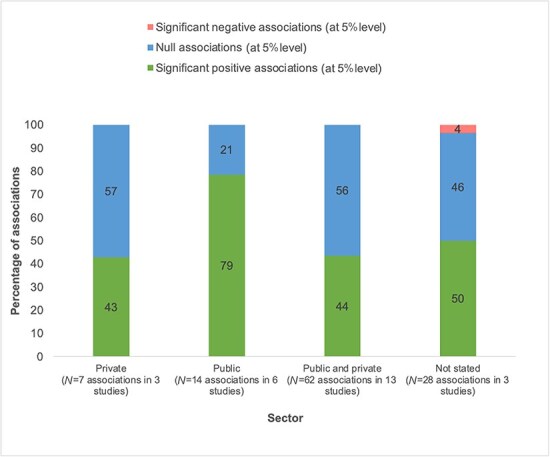
Management–quality association, by sector.

A small number of management–quality associations (21 out of 111) were tested in a single sector (private for-profit or not-for-profit) or public. Among studies conducted in the public sector only, 11 out of 14 (79%) associations are significantly positive. For those conducted in the private sector only, three out of seven (43%) associations are significantly positive (Fig. 5).

We compared studies that had used the World Management Survey approach with those that adapted the approach or used a different approach. Among studies using the World Management Survey approach, 12 out of 14 (85.7%) associations are significantly positive compared to 14 out of 37 (37.8%) associations in studies that adapted the World Management Survey and 29 out of 60 (48%) associations in studies using a different approach (Figure A1 in the Supplementary material).

## Discussion

We reviewed the global evidence on the association between adoption of management practices and quality of care provided in hospitals. The 25 studies included in this review analyzed a total of 111 associations. Overall, evidence on the association between adoption of management practices and quality of care was mixed: there was a similar proportion of associations that were significantly positive compared to those that were null. Evidence from studies conducted in high-income settings did not differ from those in low- and middle-income settings. The majority of associations were significantly positive between management practices and structural quality, clinical quality, and health outcomes. By contrast, associations between management practices and patient satisfaction or experience with care were mostly null. Notably, all studies had at least a moderate risk of bias. Indeed, those studies with a serious risk of bias had a higher proportion of significantly positive associations compared to those with a moderate risk of bias.

We consider the results on the association between hospital management practices and clinical quality of care to be the most policy-relevant aspect. These results address whether management can potentially improve provider behaviour, the efficiency of health system performance, and patient care. Studies measured clinical quality using various methods: direct observation, standardized patients, clinical vignettes, and clinical records. Each method has its advantages and disadvantages (Peabody et al. 2000, King et al. 2019), and there was no evidence that the choice of method affected the direction of the association. Studies that examined the association between management practices and health outcomes are also valuable when there is complementary data on clinical quality because they give a more complete picture of how management leads to improvement in patient health. In this review, among the 13 studies that measure health outcomes, only two (Mcconnell et al. 2013, Byabagambi et al. 2017) have complementary data on clinical quality.

The findings on structural quality refer to the availability of inputs such as drugs and equipment, which are prerequisites for good clinical care. These findings are likely to be particularly relevant in low-resource settings. However, we note that only a limited number of studies contribute to this evidence.

There was limited evidence of a positive association between management practices and quality of care, measured by patient satisfaction. In this review, the four studies that measured outcomes from the patient’s perspective (Groene et al. 2015, Macarayan et al. 2019, Kim et al. 2022, Wang et al. 2022) asked the patient to rate their satisfaction with elements of their care. The limited association with these outcomes may be driven, in part, by the fact that management processes are configured towards improving clinical care rather than being focused on patient experience. Additionally, from a methodological perspective, self-reported patient satisfaction and experience of care are notoriously difficult to measure reliably due to the range of potential factors that influence a patient’s perception of the care they receive such as individual experience and expectations of care, processed information, and rumour (Hanefeld et al. 2017). Responses tend to be overwhelmingly positive in LMICs due to survey methodology and low expectations among patients (Dunsch et al. 2018).

Understanding whether the management–quality association differs between public and private sector hospitals has an important bearing on policy, as the levers available to governments may vary by sector. In the public sector, governments may be able to intervene in management directly or by promoting internal market competition (Bloom et al. 2015b). In the private sector, governments may be able to use purchasing mechanisms to drive changes in management performance. There may also be differences in the degree of autonomy between public and private facilities. Public hospitals are likely to have less autonomy than private facilities influencing important aspects of management such as a hospital’s ability to hire new staff and their financial resources. Seven of the eligible studies compared management scores between hospitals in the public and private sectors with mixed results: three studies found no difference (Macarayan et al. 2019, Tsai et al. 2015, Thatte and Choi 2014), three studies found higher management scores in privately run facilities (Yoo et al. 2019, Bloom et al. 2020, Kim et al. 2022), and one study found higher management scores in publicly run facilities (Acharya et al. 2022). Potential reasons for these trends were variable and often context specific.

Our findings contribute to the broader literature on the links between organizational factors and patient care in hospitals. Closely related but distinct from hospital management, organizational culture and leadership are important but challenging avenues of research. Organizational culture in healthcare has been found to have a positive influence on quality of care (Braithwaite et al. 2017, Mannion and Davies 2018) and is inextricably linked to management because the organizational culture within which a manager works can have a critical bearing on their agency to implement change in an organization. Another interesting but challenging avenue for further research is to investigate the association between hospital leadership and quality of care (Fulop and Ramsay 2019). This would expand on a closely linked area of research that investigates the phenomenon of clinicians as leaders and the potential they have to positively influence hospital performance outcomes (Sarto and Veronesi 2016).

We found three intervention studies that met our inclusion criteria and only one of these was a randomized controlled trial. The vast majority of eligible studies used a cross-sectional study design and minimized the potential for confounding at the analysis stage. A challenge of this is that for some variables, such as patient load or staff management qualification, it is difficult to judge whether they are a confounder or on the causal pathway. Other studies adjusted for confounders during data collection. Certain methods for measuring clinical quality, such as using standardized patients who anonymously visit healthcare providers and document their care experience without the providers’ knowledge, address this issue by ensuring that potential confounders such as unobserved patient attributes are held constant by design (Kwan et al. 2019).

Management is a difficult construct to define and measure quantitatively. The fact that we found a sizeable literature suggests that progress has been made on this front. Many of the studies included in this review use or adapt the World Management Survey (Bloom and Van Reenen 2007) to measure management quantitatively. It aims to minimize subjectivity by using widely accepted definitions of ‘good’ and ‘bad’ management for each practice and uses a discrete scale to score participants’ responses. An inherent challenge of quantifying management practices in this way is that the execution of these practices could be variable. For example, while there is wide consensus that regular staff appraisals are an important tool, there will be considerable differences in terms of impact depending on how skilled, knowledgeable, and supportive the appraiser is.

One of the strengths of our review was the application of ASReview, an active learning software. In our process, two researchers screened the top 10% of articles prioritized by the software, while one researcher reviewed the remaining 90%. This approach allowed us to potentially save time by double screening only 10%. We also created a more expansive search strategy with broader search concepts. ASReview successfully identified all eligible studies within the top 10% of prioritized articles, demonstrating its potential for future systematic reviews, especially when dealing with complex and abstract concepts commonly found in the field of health systems research.

Another strength is that we conducted a global review of evidence, making the findings more widely applicable and allowing us to comment on whether the management–quality relationship differs between different income settings. We also categorized the quality of care outcomes to deal with heterogeneity and improve the interpretation of findings.

The study had several limitations. First, our review, with its focus on management practices, speaks to the quality of management, but not the quantity of management, by which we mean the optimal number of managers and how they are distributed at different levels of the health system. Secondly, we did not search the grey literature and could have missed publications about the management–quality association that are not peer-reviewed research such as reports from nongovernmental or governmental organizations, consultancies, or United Nations (UN) bodies. Finally, due to our broad search terms and inclusive eligibility criteria, the included studies are heterogenous in terms of management measures and outcomes, making it challenging to directly compare the management–quality coefficients between studies.

This systematic review demonstrates that hospital management practices can be evaluated, making it a dimension of hospital performance that policymakers may wish to track over time. It provides mixed evidence on the relationship between management practices and quality of care, presenting some evidence of association in certain settings. We believe the findings, particularly those on the association between management practices and clinical quality of care, warrant further investigation through intervention studies or, where opportunities arise, natural experiments. Ideally the former would use a randomized design, measure management practices with a validated tool, and leverage high-quality routine information systems to measure quality of care. However, a key challenge is to conceptualize and develop an intervention to enhance hospital management in a way that will positively impact quality of care and health outcomes. This could build on partial attempts that have been made to define management interventions in the education sector in LMICs (Karthik and Abhijeet 2020). It will likely also require a more in-depth elucidation of the causal pathways between management and health outcomes. Our understanding of these relationships will benefit from development in defining and measuring intermediary outcomes to trace how management practices influence patient health. Qualitative health systems research could also help open up this ‘black box’ (Marchal et al. 2010), with additional insights about strategies to improve quality of care provided by the burgeoning quality improvement literature (Wells et al. 2018, Bircher 2019). In the meantime, policymakers may do well to consider and debate how government and hospital leaders can address poor management in areas that are most plausibly influential for patient care.

## Supplementary Material

czae112_Supp

## Data Availability

All data are available in the tables presented in this publication.

## Reflexivity statement

The authors include five females and three males, spanning multiple levels of seniority. The cross-disciplinary team brings expertise from health economics and management, epidemiology, implementation science, social science and health policy. The team also includes medical practitioners who have held management positions in hospitals. This systematic review of global evidence has benefited from the collaboration between the authors from Malawi and the UK who contribute diverse experience and knowledge on the topic of hospital management and quality of care.
