# Supplementary material for: What is the relationship between hospital management practices and quality of care? A systematic review of the global evidence
Source: Health Policy Plan. 2024 Nov 22;40(3):409–21. doi: 10.1093/heapol/czae112 (PMC11886796; doi:10.1093/heapol/czae112)
Supplement: czae112_Supp [file czae112_supp.zip › Supplementary file_080125.docx]

# Supplementary File

Database searches were carried out on 1 August 2022 and 16 October 2024.

Table A1 Search strategy for PubMed database

| **No.** | **Search terms** |
| --- | --- |
| Terms: Title and abstract  With applied NCBI filters: Date range 01/01/2000 onwards.  Text availability “abstract” | |
| 1 | “Hospital management” OR  “Health management” OR  “Management capacity” OR  “Management practice*” OR  “Management performance” OR  “Management score*” OR  “Management tool” OR  “Management scorecard” OR  “Health care management” OR  “Healthcare management” OR  “Health-care management” OR  “Health service management” |
| 2a | “Quality of care” OR  “Quality of health care” OR  “Quality of healthcare” OR  “Health care quality” OR  “Healthcare quality” OR  “Care quality” OR  “Clinical standard*” OR  “Clinical outcome*” OR  “Health outcome*” OR  “Structural quality” OR  “Process quality” OR  “Hospital performance” OR  “Clinical performance” |
| 2b | “Quality of health care” [Mesh] |
| 3 | Hospital* OR  Department* OR  Care OR  Ward OR  Provider* OR  Unit |
| 4 | 2a OR 2b |
| 5 | 1 AND 3 AND 4 |

Table A2 Search strategy for EMBASE and Global Health databases

| **No.** | **Search terms** |
| --- | --- |
| Search fields: title and abstract  Limits: 01-01-2000 to current  Limits: abstracts | |
| 1 | Hospital management OR  Health management OR  Management capacity OR  Management practice* OR  Management performance OR  Management score* OR  Management tool OR  Management scorecard OR  health care management OR  healthcare management OR  health-care management OR  health service management |
| 2a | Quality of care OR  quality of health care OR  quality of healthcare OR  health care quality OR  healthcare quality OR  care quality OR  clinical standard* OR  clinical outcome* OR  health outcome* OR  structural quality OR  process quality OR  hospital performance OR  clinical performance |
| 2b | Exp “quality of health care” [Map term to subject heading] |
| 3 | Hospital* OR  Department* OR  Care OR  Ward OR  Provider* OR  Unit |
| 4 | 2a OR 2b |
| 5 | 1 AND 3 AND 4 |

Table A3 Search strategy for EconLit database

| **No.** | **Search terms** |
| --- | --- |
| Search fields: title and abstract  Limits: 01-01-2000 to current  Limits: abstracts | |
| 1 | Hospital management OR  Health management OR  Management capacity OR  Management practice* OR  Management performance OR  Management score* OR  Management tool OR  Management scorecard OR  health care management OR  healthcare management OR  health-care management OR  health service management |
| 2 | Quality of care OR  quality of health care OR  quality of healthcare OR  health care quality OR  healthcare quality OR  care quality OR  clinical standard* OR  clinical outcome* OR  health outcome* OR  structural quality OR  process quality OR  hospital performance OR  clinical performance |
| 3 | Hospital* OR  Department* OR  Care OR  Ward OR  Provider* OR  Unit |
| 4 | 1 AND 2 AND 3 |

Table A4 Search strategy for Web of Science database

| **No.** | **Search terms** |
| --- | --- |
| Search fields: title and abstract  Limits: 01-01-2000 to current  Limits: abstracts | |
| 1 | “Hospital management” OR  “Health management” OR  “Management capacity” OR  “Management practice*” OR  “Management performance” OR  “Management score*” OR  “Management tool” OR  “Management scorecard” OR  “Health care management” OR  “Healthcare management” OR  “Health-care management” OR  “Health service management” |
| 2 | “Quality of care” OR  “Quality of health care” OR  “Quality of healthcare” OR  “Health care quality” OR  “Healthcare quality” OR  “Care quality” OR  “Clinical standard*” OR  “Clinical outcome*” OR  “Health outcome*” OR  “Structural quality” OR  “Process quality” OR  “Hospital performance” OR  “Clinical performance” |
| 3 | Hospital* OR  Department* OR  Care OR  Ward OR  Provider* OR  Unit |
| 4 | 1 AND 2 AND 3 |

Table A5 Criteria for assessing a study’s Risk of Bias. Adapted from ROBINS-I (Sterne et al., 2016)

| **Risk of bias judgement** | **Domain 1 - confounding*** | **Domain 2 - participant selection** | **Domain 3 - classification of management** | **Domain 4 - deviation from intervention** | **Domain 5 - Missing data** | **Domain 6 - Outcome measure** | **Domain 7 - selection of reported result** |
| --- | --- | --- | --- | --- | --- | --- | --- |
| **Low** | Study uses a randomised design; study uses longitudinal data and relevant confounders are controlled for with an appropriate analysis method; confounders were measured reliably and validly. | Study sampled all possible hospitals (e.g. through census); study used a systematic sampling strategy with a high response rate. | Study shows conceptual rigour in how they have defined management practices and, if applicable, their overall summary measure; the tool to measure management practices is developed or adapted from published tools; management practices are measured in a way that limits bias. | There was no deviation from the intervention beyond what would be expected in usual practice; the intervention was implemented successfully for all participants; participants adhered to the assigned intervention. | No missing data reported for outcome or exposure data; study uses routinely collected data from a high quality, established information system. | Study uses routine data from a high quality information system; outcomes are defined from established measures; study uses systematically collected primary data based on validated tools. The reliability of the outcome measure does not vary according to strength of hospital management practices. | Study pre-specified the analysis or registered the study; study has clearly defined and well-described primary and secondary outcomes; study conducts robustness checks around the main findings; study conclusion matches the reported findings. |
| **Moderate** | Study uses a cross-sectional design and controls for relevant confounders with an appropriate analysis method; confounders were measured reliably and validly. | Study used a systematic sampling strategy but had a moderate response rate; study had a lower response rate but demonstrated that their sample is representative of the population of interest for key variables. | There is some level of conceptual rigour in how the study defines management practices and, if applicable, their overall summary measure. | There was some deviation from intervention; the intervention was implemented successfully for most participants. | Some degree of missing data reported for outcome or exposure data. Missing data does not differ between hospitals with different strengths of management practices. | Study uses routine data from a moderate quality information system; study uses systematically collected primary data but without validation. There is a clear rationale for outcomes chosen and a clear description of how they are measured. | Primary and secondary outcomes are specified in the methods section but lack thorough description of rationale for selection. |
| **Serious** | Study uses cross sectional design with poorly measured or defined confounders or does not adjust for confounders. | Study used a non-systematic sampling strategy; study had a low response rate. | There is little rigour in the conceptualisation of different management practices and their overall summary measure; variables are chosen ad hoc. | There was some deviation from intervention; the intervention was implemented unsuccessfully. | High degree of missing data reported for outcome or exposure data. | Study uses routine data from a poor quality information system; study uses primary data collected with no methodological rigour or with large scope for bias. | Study conducts multiple analyses e.g. for different types of outcomes, for different sub-groups or different exposure-outcome relationships without clear rationale or prioritisation of primary/secondary outcomes. No robustness checks around the main findings. |
| **Critical** | n/a | Study used a very small sample size (<10) and purposefully selected hospitals. | Nonsensical concept of management practices. | There was major deviation from the intervention; the intervention was implemented unsuccessfully. | Very high degree of missing data reported for outcome or exposure data. Missing data varies between hospitals with different strengths of management practices. | Source of data for the outcome measures are not specified. No conceptual rigour in the selection or definition of the outcomes. | Study reports results in an ad hoc manner without rationale or explanation. No robustness checks around the main findings. |
| *Appropriate confounders include:  Hospital size and capacity (e.g. number of patients, catchment area, total budget, number of staff);  Type of hospital (e.g. public/private/mission; teaching hospital); Patient characteristics (e.g. case-mix; age, sex) | | | | |  |  |  |

Table A6 Risk of Bias Assessment. Adapted from ROBINS-I (Sterne et al., 2016)

| **Study** | | **Domain 1 - confounding*** | **Domain 2 - participant selection** | **Domain 3 - classification of management** | **Domain 4 - deviation from intervention** | **Domain 5 - Missing data** | **Domain 6 - Outcome measure** | **Domain 7 - selection of reported result** | **Overall** |
| --- | --- | --- | --- | --- | --- | --- | --- | --- | --- |
| Acharya et al (2022) | | Moderate: cross-sectional study with appropriate confounders controlled for | Low: systematic sampling strategy and low level of non-response | Moderate: authors select four management-related items from a broader health facility survey | Not applicable | Low: no reported missingness | Low: secondary data from the 2015 Nepal Health Facility Survey and used World Health Organization service readiness manual | Low: pre-specified analysis and robustness checks performed | Moderate |
| Adhikari et al (2024) | | Moderate: Cross-sectional, relevant confounders controlled for | Low: national-level survey sample | Moderate: authors select four management-related items from a broader health facility survey | Not applicable | Low: no reported missingness. | Low: secondary data from the 2021 Nepal Health Facility Survey | Low: pre-specified analysis | Moderate |
| Adler-Milstein et al (2014) | | Moderate: cross-sectional study with appropriate confounders controlled for | Moderate: authors demonstrated that the analytic sample is similar in terms of key hospital characteristics, to a broader hospital organisation sample (American Hospital Association) | Low: management was measured using the WMS | Not applicable | Low: study uses data from an established information system - the American Hospital Association annual survey | Low: data from an established information system - the American Hospital Association annual survey; established AMI outcomes are used; AMI selected because the majority of interviewees work in cardiology units | Low: pre-specified analysis | Moderate |
| Asaria et al (2021) | | Moderate: cross-sectional study with appropriate confounders controlled for | Low: study uses a national sample of NHS acute care hospitals | Serious: management measured using staff members' perception of their immediate and senior managers | Not applicable | Low: study uses data from an established, routine information system called NHS digital | Low: data from a routine information system called NHS digital | Low: analyses were pre-specified; there is consistent reporting of the exposure-outcome relationships for each type of outcome and each type of model (with and without size and case-mix controls); robustness checks performed | Serious |
| Bloom et al (2015b) | | Moderate: cross-sectional study with appropriate confounders controlled for | Moderate: 61% response rate | Low: management was measured using the WMS | Not applicable | Low: study uses data from an established information system | Low: administrative datasets from high quality information systems are the sources for outcome data. Mortality from emergency AMI and Mortality rate from emergency surgery use Hospital Episode Statistics data; Healthcare Commission rating is from the Commission for Healthcare Audit and Inspection responsible for assessing standards of care provided by the NHS. | Moderate: analyses are pre-specified but there is no clear prioritisation | Moderate |
| Bloom et al (2020) | | Moderate: cross-sectional study with appropriate confounders controlled for | Moderate: 35% response rate. Authors compare their sample to all eligible hospitals and are aware of the biases in their sample. | Low: management was measured using the WMS | Not applicable | Low: study uses data from an established information system | Low: data are from publicly available datasets from established information systems | Moderate: analyses are pre-specified but there is no clear prioritisation | Moderate |
| Byabagambi et al (2017) | | Serious: before and after study which compares baseline and endline compliance with improvement indicators. No confounders are adjusted for. | Serious: study was implemented in three purposefully selected districts and health facilities. | Serious: study lacks a clear rationale in the selection of management items to measure | Moderate: the intervention was implemented as intended with some delays | Moderate: authors excluded some data due to missingness. | Moderate: outcome data collected from primary data sources (record review and interviews with clients and health facility staff) | Moderate: analyses are pre-specified but there is no clear prioritisation | Serious |
| Fanelli et al (2020) | | Moderate: cross-sectional study with appropriate confounders controlled for | Moderate: study sites were not selected systematically, rather they volunteered to be part of a wider project called Italian Neonatal Network. The sample represents 43% of neonatal intensive care units nationally. Authors demonstrate that the sample is representing a high proportion of cases nationally. High response rate among interviewees (>96%) | Moderate: authors define five expansive areas of management within which managers make choices. They are selected from a broader framework without clear explanation. | Not applicable | No information | Moderate: outcome data collected from primary data sources using cross-sectional survey in the neonatal intensive care unit | Low: pre-specified analysis. | Moderate |
| Groene et al (2015) | | Moderate: cross-sectional study with appropriate confounders controlled for | Moderate: study used a systematic sampling strategy; analytical sample was comparable to the overall sample for hospital characteristics, response rate 75-100%; low response rate in 1 country (4 hospitals in Germany) | Low: authors use a multi-dimensional measure of management that covers relevant domains and is based on previously validated tools (WMS) | Not applicable | Low: low levels of missing data | Low: outcome data are collected from previously validated tools. | Low: pre-specified analysis. | Moderate |
| McConnell et al (2013) | | Moderate: cross-sectional study with appropriate confounders controlled for | Moderate: some differences in the baseline characteristics between those who did and did not respond | Low: management was measured using the WMS | Not applicable | Low: no missing data reported | Low: secondary data collected through routine government information systems for an insurance provider (Medicare) | Low: pre-specified analysis. | Moderate |
| Mwencha et al (2017) | | Moderate: before and after study which compares baseline and endline data on logistics management. | Serious: non-systematic sampling strategy | Serious: little conceptual rigour in classification of management | No information | No information | Moderate: primary data collection without validation | Low: pre-specified analysis. | Serious |
| Mwihia et al (2019)^ | | Serious: cross-sectional study with no adjustment for confounders | Low: study sampled all 25 hospitals in central Kenya | Serious: no rationale given for the management practices that are selected; management is not measured using established or validated tools | Not applicable | No information | Critical: paper does not describe how the outcome measures are generated | Serious: primary and secondary outcomes are not specified, there are no robustness checks | Critical: exclude |
| Plough et al (2017) | | Moderate: cross-sectional study with appropriate confounders controlled for | Serious: non-systematic sampling strategy | Low: authors use a multi-dimensional measure of management that covers relevant domains and is based on previously validated tools (WMS) | Not applicable | No information | Low: study uses data from an established information system | Low: pre-specified analysis. | Serious |
| Powell-Jackson et al (2024) | | Moderate: cross-sectional study with appropriate confounders controlled for | Low: authors approached all eligible facilities in Tanzania and had a high response rate. | Low: authors use a multi-dimensional measure of management that covers relevant domains and is based on previously validated tools (WMS) | Not applicable | Low: low levels of missing data | Low: primary data collection with standardised patients using established tools. | Low: pre-specified analysis | Moderate |
| Salas-Ortiz et al (2019) | | Moderate: cross-sectional study with appropriate confounders controlled for | Low systematic sampling strategy | Low: multi-dimensional measure of management using established tools | Not applicable | No information | Low: study uses systematically collected primary data based on established tools | Low: pre-specified analysis | Moderate |
| Salehnejad (2022) | | Moderate: cross-sectional study with appropriate confounders controlled for | Low: national-level survey sample | Serious: management measured using staff members' perception of their immediate and senior managers | Not applicable | Low: low levels of missing data | Low: data from a routine information system called NHS digital | Low: pre-specified analysis | Serious |
| Thatte et al (2014) | | Moderate: cross-sectional study with appropriate confounders controlled for | Low: national-level survey sample | Serious: 3 items of HR management selected from a wider survey, little conceptual rigour | Not applicable | No information | Low: primary data collection with observation and record review | Low: pre-specified analysis | Serious |
| Tsai et al (2015) | | Moderate: cross-sectional study with appropriate confounders controlled for | Moderate: study sites were selected at random with a moderate response rate | Low: management was measured using the WMS | Not applicable | No information | Low: study uses data from an established information system | Low: pre-specified analysis | Moderate |
| Wang et al (2022) | | Moderate: cross-sectional study with appropriate confounders controlled for | Serious: study sites were not selected systematically; low response rate. Authors did not demonstrate similarity in characteristics between study and non-study hospitals | Low: management was measured using the WMS | Not applicable | Moderate: some missing data | Moderate: primary data collection of patient satisfaction data | Low: prespecified analysis | Serious |
| West et al (2002) | | Moderate: cross-sectional study with appropriate confounders controlled for | Moderate: authors demonstrate similarity in those participating compared to those not participating | Moderate: authors demonstrate some conceptual rigour in how they define the areas of management. Not measured through established tools | Not applicable | No information | Moderate: primary data collection | Moderate: analyses are pre-specified but there is no clear prioritisation | Moderate |
| Yoo et al (2019) | | Moderate: cross-sectional study with appropriate confounders controlled for | Moderate: purposive site selection | Low: management was measured using the WMS | Not applicable | Low: low levels of missing data | Low: study uses systematically collected primary data based on established tools. | Low: pre-specified analysis | Moderate |
| Zhu et al (2021) | | Moderate: cross-sectional study with appropriate confounders controlled for | Moderate: study sites selected purposefully as part of a wider randomised controlled trial | Low: authors use a multi-dimensional measure of management that covers relevant domains and is based on previously validated tools (WMS) | Not applicable | Moderate: study reports some missing data that are handled using the linear mixed model | Serious: rationale for the selected indicators and how they are measured is not described | Low: pre-specified analysis | Serious |
| King et al (2021) | | Low: cluster randomised controlled trial | Low: authors approached all eligible facilities in Tanzania and had a high response rate. | Low: established SafeCare tool used to measure management practices | Moderate: intervention fidelity was lower than expected for some elements of the intervention. | Low | Low: study uses systematically collected primary data based on validated tools | Low: pre-specified analysis | Moderate |
| West et al (2006) | | Moderate: cross-sectional study with appropriate confounders controlled for | Low: authors checked for and found no non-response bias | Low: authors use a multi-dimensional measure of management that covers relevant domains based on literature search | Not applicable | Moderate: study reports some missing data | Low: study uses data from an established information system | Low: pre-specified analysis | Moderate |
| Pollack et al (2003) | | Moderate: cross-sectional study with appropriate confounders controlled for | Critical: small sample size (<10) of purposefully selected facilities | Low: authors use a multi-dimensional measure of management that covers relevant domains based on literature search | Not applicable | Moderate: study reports some missing data | Low: study uses data from an established information system | Low: pre-specified analysis | Critical: exclude |
| Macarayan et al (2019) - structural outcome | | Moderate: cross-sectional study with appropriate confounders controlled for | Low: health facilities and households were surveyed in a systematic way and were nationally representative | Low: authors use a multi-dimensional measure of management that covers relevant domains and is based on previously validated tools (WMS) | Not applicable | Moderate: some missing data | Low: indices systematically collected and based on established tools | Low: pre-specified analysis. | Moderate |
| Macarayan et al (2019) - patient satisfaction outcome | | Moderate: cross-sectional study with appropriate confounders controlled for | Low: health facilities and households were surveyed in a systematic way and were nationally representative | Low: authors use a multi-dimensional measure of management that covers relevant domains and is based on previously validated tools (WMS) | Not applicable | Low: no missing data reported | Moderate: primary data collection of experiential data without validation | Low: pre-specified analysis. | Moderate |
| Kim et al (2022) - structural outcome | | Moderate: cross-sectional study with appropriate confounders controlled for | Low: health facilities and households were surveyed in a systematic way using the National Census sampling frame | Low: authors use a multi-dimensional measure of management that covers relevant domains and is based on previously validated tools (WMS) | Not applicable | Low: low levels of missing data | Low: indices systematically collected and based on established tools | Low: pre-specified analysis. | Moderate |
| Kim et al (2022) - patient satisfaction outcome | | Moderate: cross-sectional study with appropriate confounders controlled for | Low: health facilities and households were surveyed in a systematic way using the National Census sampling frame | Low: authors use a multi-dimensional measure of management that covers relevant domains and is based on previously validated tools (WMS) | Not applicable | Low: low levels of missing data | Moderate: primary data collection of experiential data without validation | Low: pre-specified analysis. | Moderate |
| *Appropriate confounders include: Hospital size and capacity (e.g. number of patients, catchment area, total budget, number of staff); Type of hospital (e.g. public/private/mission; teaching hospital); Patient characteristics (e.g. case-mix; age, sex)  ^Mwihia FK, M’imunya JM, Mwabu G et al. Effects of management practices on hospital outcomes in Kenya. East Afr Med J2019;95:1344–1355. | | | | | | | | |  |
| WMS: World Management Survey  AMI: acute myocardial infarction  NHS: National Health Service | | | | | | | | |  |

Table A7 Summary of eligible studies

| **Author (year)** | **Study design, number of facilities, income setting,** | **Country** | **Sector (private (for-profit or not-for profit), public** | **Setting** | **Coefficient and sub-group estimates** | **Measure of spread** | **Interpretation** | **Total # of associations** | **Total # of significant (p<0.05) associations in expected direction** | **Total # of significant (p<0.05) associations in unexpected direction** | **Proportion of associations significantly, positive (at 5 percent level)? All / majority / minority / none** |
| --- | --- | --- | --- | --- | --- | --- | --- | --- | --- | --- | --- |
| Acharya et al (2022) | Cross-sectional, 940 facilities, LMIC | Nepal | Public and private | Hospitals, health centres, health posts, clinics | Service readiness in health facilities with: 1) Regular management meetings: 2.98%* 2) External supervision in last 4 months: 4.00%* 3) Routine quality assurance activities performed: 2.02%* 4) System of collecting opinion and reviewed: 4.71%* | SE: 1) 1.18 2) 0.94 3) 1.06 4) 2.12 | Health facilities with regular management meetings had a 2.98% increase in service readiness compared to those without regular management meetings | 4 | 4 | 0 | All positive |
| Adhikari (2024) | Cross-sectional, 1535 facilities, LMIC | Nepal | Public and private | Hospitals, health centres, health posts, clinics | Service readiness in health facilities with:  1) Quality assurance performed: 3.68%** 2) External supervision - yes: 0.16% 3) System to take client opinion - yes: 6.66%* 4) Have a monthly health facility meeting: 3.28%* | 95% CI: 1) 1.84-5.51 2) -1.81-1.48 3) 2.54-10.77 4) 1.08-5.49 | Health facilities with quality assurance performed had a 3.68% higher readiness score compared to those that did not have quality assurance performed | 4 | 4 | 0 | All positive |
| Adler-Milstein et al (2014) | Cross-sectional, 191 facilities, HIC | US | Public and private | Hospitals | 30-day mortality: -0.04 | SE=0.04 | No evidence of an association between management score and 30-day mortality | 1 | 0 | 0 | All null |
| Asaria et al (2021) | Cross-sectional, 129 facilities, HIC | England | Public | Hospitals | Summary hospital-level mortality indicator: 0.0001 | SE=0.001 | No evidence of an association between quality of management and mortality | 1 | 0 | 0 | All null |
| Bloom et al (2015b) | Cross-sectional, 161 facilities, HIC | England | Public | Hospitals | 1) Mortality rate from emergency AMI: -0.968*  2) Mortality rate from all emergency surgery: -0.099* 3) Health Care Commission performance: 0.108** | SE:  1) 0.481 2) 0.044 3) 0.041 | A one SD increase in the overall management score of public hospitals was associated with a reduction of 0.97 percentage points in the mortality rate from AMI | 3 | 3 | 0 | All positive |
| Bloom et al (2020) | Cross-sectional, 478 facilities, HIC | US, UK, Sweden, Germany, Canada, Italy, France, Brazil | Public and private | Hospitals | AMI death rates: -0.188** | SE=0.055 | A one SD increase in a hospital’s management score is associated with a fall of −0.188 SD in AMI deaths rates | 1 | 1 | 0 | All positive |
| Byabagambi et al (2017) | Before and after intervention study, 14 facilities, LMIC | Uganda | Not stated | Hospitals, health centres | 1) % clients demonstrating good clinical wellness: +28%** 2) % clients with >95% adherence to ARV: +20%** 3) % clients collecting medicines as scheduled: -6%** (significant negative association) 4) Client was given the prescribed medicine: +6%** 5) Dispensed medicine labelled: +2% 6) If labelled, correct label?; +13%** 7) Medication dosage included; +1% 8) Correct dosage included?; +9%** 9) Medication quantity included; +41%** 10) No labelling errors/omissions; +45%** 11) Client knows why he/she is getting medicine?; -8% 12) If labelled, client can read medicine label? +23%** 13) Client can explain details of therapeutic course? +8% | Not presented | The percentage of clients demonstrating good clinical wellness increased by 28% between baseline and endline | 13 | 8 | 1 | Majority |
| Fanelli et al (2020) | Cross-sectional, 51 facilities, HIC | Italy | Not stated | Hospital neonatal intensive care units | Authors tested for differences between managerial models on the outcomes below. Three managerial models were a) traditional; b) collaborative; c) individualistic. 1) Mortality: a) 14.97*; b) 12.01*; c) 14.85 2) Nosocomial infection: a) 14.76; b) 13.81; c) 14.41 3) Severe intraventricular haemorrhage: a) 8.97; b) 7.41; c) 8.89 4) Severe Retinopathy of Prematurity: a) 8.10; b) 7.03; c) 8.56 5) Morbidity: a) 39.02; b) 36.76; c) 38.93 6) Necrotizing Enterocolitis: a) 4.12; b) 4.25; c) 3.93 7) Periventricular leukomalacia: a) 5.17; b) 4.79*; c) 6.70* 8) Pulmonary Bronchodysplasia: a) 20.81; b) 19.39; c) 19.01 9) Human milk: a) 62.39; b) 63.36; c) 61.64 | SD: 1a) 2.66 1b) 4.52 1c) 4.86 2a) 1.80 2b) 3.52 2c) 3.68 3a) 1.93 3b) 3.11 3c) 3.89 4a) 1.69 4b) 3.52 4c) 4.40 5a) 4.79 5b) 8.64 5c) 8.64 6a) 0.78 6b) 0.82 6c) 1.04 7a) 1.74 7b) 1.21 7c) 2.55 8a) 3.07 8b) 6.09 8c) 7.16  9a) 1.57 9b) 2.94 9c) 3.62 | There is a significant difference in the mortality rate between managerial models (a) and (b) | 9 | 2 | 0 | Minority |
| Groene et al (2015) | Cross-sectional, 74 facilities, HIC | Czech Republic, France, Germany, Poland, Portugal, Spain, Turkey | Public and private | Hospitals | 1) Patient experience: a) AMI: 0.4; b) Deliveries: 0.11; c) hip fracture: -0.4; d) stroke: 0.13 2) Patient perceived involvement in care: a) AMI: 0.02; b) Deliveries: 0.00; c) hip fracture: -0.03; d) stroke: 0.0 3) Patient healthcare transition score: a) AMI: 0.18; b) Deliveries: 0.05; c) hip fracture: -0.43; d) stroke: 0.19 4) Patient will recommend hospital: a) AMI: 0.02*; b) Deliveries: 0.00*; c) hip fracture: -0.02; d) stroke: -0.00 | SE: 1a) 0.24 1b) 0.21 1c) 0.29 1d) 0.26 2a) 0.02 2b) 0.01 2c) 0.02 2d) 0.01 3a) 0.32 3b) 0.23 3c) 0.23 3d) 0.27 4a) 0.01 4b) 0.01 4c) 0.01 4d) 0.01 | There is a significant relationship between quality management system index and whether the patient will recommend the hospital for two care pathways | 16 | 2 | 0 | Minority |
| Kim et al (2022) | Cross-sectional, 287 facilities, LMIC | Uganda | Public and private | Hospitals, health centres and health clinics | 1) Essential drug index: a) quintile 1=0.67; b) quintile 5 =0.75** 2) Equipment index: a) quintile 1=0.81; b) quintile 5=0.99 3) Prompt attention (waiting time): 1.49 4) Facility cleanliness: 2.40 5) Trust in providers: 1.26 6) Respect rating: 0.66 7) Ease of understanding provider's advice: 1.32 8) Ease of following provider's advice: 1.14 9) Likelihood of returning to the facility: 1.22 10) Quality rating: 1.25 | 95% CI: 1) 0.60-0.74; b) 0.70-0.79 2) 0.73-0.89; b) 0.96-1.01 3) 0.39-5.65 4) 0.97-5.96 5) 0.83-1.91 6) 0.23- 1.93 7) 0.47-3.68 8) 0.72-1.82 9) 0.86-1.73 10) 0.35-4.47 | Ugandan health facilities in the highest management quintile had essential drug index scores of 0.75 compared to 0.67 in the lowest management quintile | 10 | 1 | 0 | Minority |
| King et al (2021) | Cluster randomised controlled trial, 228 facilities, LMIC | Tanzania | Private | Dispensaries, health centres, hospitals | 1) Infection prevention and control compliance: 1.10 2) Correct case management: 0.87 | 95% CI: 1) 0.99-1.21 2) 0.65-1.17 | Odds of infection prevention and control compliance in the control versus intervention group was not significantly different | 2 | 0 | 0 | All null |
| Macarayan et al (2019) | Cross-sectional, 142 facilities, LMIC | Ghana | Public and private | Hospitals or polyclinic, health centres, clinics, community health planning and services | 1) Essential drug index: 1.22** 2) Equipment index: 1.04 3) Prompt attention (waiting time): 0.78* 4) Facility cleanliness: 1.10 5) Trust in providers: 1.08* 6) Respect rating: 1.05 7) Ease of understanding provider's advice: 1.03 8) Ease of following provider's advice: 1.15* 9) Likelihood of returning to the facility: 1.00 10) Quality rating: 1.16* | 95% CI: 1) 1.07-1.37 2) 0.99-1.08 3) 0.61-0.99 4) 0.96-1.25 5) 1.01-1.16 6) 0.90-1.22 7) 0.95-1.11 8) 1.01-1.30 9) 0.93-1.09 10) 1.02-1.32 | Facilities in the 90th percentile (management score = 0.90) had 22% more essential drugs in comparison to facilities with management scores in the 10th percentile (management score = 0.60) | 10 | 5 | 0 | Majority |
| McConnell et al (2013) | Cross-sectional, 597 facilities, HIC | US | Public and private | Hospitals | 1) 30-day risk adjusted mortality rate: -0.17* 2) Aspirin use within 24 h of arrival: 0.06* 3) ACEI use for left ventricular dysfunction: 1.6* 4) Provision of percutaneous coronary intervention within 90 min of arrival: 1.6* 5) Aspirin prescribed at discharge: 0.08* 6) Blocker prescribed at discharge: 0.16* 7) Provision of smoking cessation counselling: 0.9* 8) 30-day risk adjusted readmissions: 1.02 | 95% CI: 1) -0.31- -0.05 2) 0.02-0.18 3) 0.7-3.4 4) 0.32-2.9 5) 0.01-0.29 6) 0.04-0.47 7) 0.05-4.2 8) 0.97-1.07 | Facilities in the 75th percentile had -0.17 difference in 30-day risk adjusted mortality rate compared to those in the 25th percentile management score | 8 | 7 | 0 | Majority |
| Mwencha et al (2017) | Before and after intervention study, 220 facilities, LMIC | Tanzania | Public | Hospitals, dispensaries, health centres | 1) -49% odds of stocking out** 2) -44% odds of stocking out for greater than 7 days** | Not presented | The odds of stocking out fell by 49% from baseline to round 1 | 2 | 2 | 0 | All positive |
| Plough et al (2017) | Cross-sectional, 51 facilities, HIC | US | Not stated | Hospitals | Predictors of quality of care by management theme: a) unit management culture; b) patient flow management; c) nursing management 1) Low-risk primary caesarean delivery: a) 1.30*; b) 1.05; c) 1.47* 2) Prolonged length of stay: a) 4.13*; b) 0.23*; c) 0.27* 3) Severe maternal morbidity: a) 1.60; b) 1.28; c) 0.77 4) Maternal obstetric infection: a) 1.31; b) 0.95; c) 0.72 5) Postpartum haemorrhage: a) 2.57*; b) 0.78; c) 1.20 6) Blood transfusion: a) 1.87*; b) 1.25; c) 0.72 | 95% CI:  1a) 1.02-1.66; 1b) 0.83-1.34; 1c) 1.13-1.92 2a) 1.98-8.64; 2b) 0.12-0.46; 2c) 0.11-0.62 3a) 0.89-2.88; 3b) 0.82-2.01; 3c) 0.45-1.33 4a) 0.83-2.06; 4b) 0.57-1.60; 4c) 0.44-1.19 5a) 1.58-4.18; 5b) 0.48-1.25; 5c) 0.75-1.92 6a) 1.12-3.13; 6b) 0.71-2.20; 6c) 0.44-1.16 | Patients who received care at the hospital with the highest unit culture management score had a 30% higher relative risk of low-risk primary caesarean delivery (RR 1.30, 95% CI 1.02-1.66) | 6 | 4 | 0 | Majority |
| Powell-Jackson et al (2024) | Cross-sectional, 220 facilities, LMIC | Tanzania | Private | Hospitals, health centres, dispensaries | 1) Correct treatment pooled: 0.29**  2) Proportion of checklist items completed: 0.10** 3) Any unnecessary care: -0.031 4) Number of antibiotics prescribed: -0.26 5) Infection prevention and control: 0.062* | SE: 1) 0.069 2) 0.038 3) 0.077 4) 0.14 5) 0.031 | A 10 percentage point increase in the management score is associated with a 2.9 percentage point improvement in correct treatment. | 5 | 3 | 0 | Majority |
| Salas-Ortiz et al (2019) | Cross-sectional, 96 facilities, LMIC | Kenya, Rwanda, South Africa, Zambia | Public and private | Hospitals, primary care clinics | Association between different types of management practices and quality of voluntary medical male circumcision: 1) Performance-based funding: 0.06  2) Sanctions: -0.1 3) External supervision: 0.03  4) Community participation: -0.09 5) National-level governance: 0.1  6) Municipal-level governance: -0.05 | 95% CI: 1) -0.03-0.16  2) -0.24-0.03 3) -0.06-0.13  4) -0.22-0.04 5) -0.01-0.22  6) -0.17-0.05 | No significant association between management practices and quality of voluntary medical male circumcision. | 1 | 0 | 0 | All null |
| Salehnejad et al (2022) | Cross-sectional, 818 facilities, HIC | England | Public | Hospitals | Management model 1:  1) -10.73** Management model 2: 2) -6.219* | Not presented | 1. Reduction in around 11 percentage points in Summary Hospital-level Mortality Indicator for hospitals with management model 1 | 2 | 2 | 0 | All positive |
| Thatte et al (2014) | Cross-sectional, 893 facilities, LMIC | Kenya | Public and private | Hospitals, health centres, maternity clinics, dispensaries, stand-alone voluntary counselling, testing clinics | Coefficient on service quality index scores by providers’ human resource management characteristics: a) Trained (no; yes); b) Received supervision (no; yes, any; yes, supportive); c) Written job description (no; yes) 1) History taking: a) 1.7; b)-3.1; c) 10.5** 2) Physical examination: a) -2.1; b) -4.7; c) 8.8** 3) STI prevention: a) 5.7; b) 0.4; c) 4.3 | Not presented | In consultations by providers with written job descriptions, quality scores for history taking and physical examination were significantly higher than their counterparts without job descriptions | 3 | 2 | 0 | Majority |
| Tsai et al (2015) | Cross-sectional, 103 facilities, HIC | US, England | Public and private | Hospitals | 1) 42.6 percent vs. 14.3 percent** | Not presented | Hospitals with management scores above the median were more likely to be high-quality hospitals (42.6 percent versus 14.3 percent) | 1 | 1 | 0 | All positive |
| Wang et al (2022) | Cross-sectional, 235 facilities, LMIC | China | Public and private | Hospitals | 1) Inpatient satisfaction: 0.463* 2) Outpatient satisfaction: 0.572 | SE: 1) 0.227 2) 0.358 | High management scores are associated with significantly higher inpatient satisfaction | 2 | 1 | 0 | Majority |
| West et al (2002) | Cross-sectional, 51 facilities, HIC | England | Public | Hospitals | Association between management and a) deaths after emergency surgery; b) deaths after hip fractures; c) mortality index 1) Sophistication of training policy: a) -0.158; b) -0.069; c) -0.306* 2) Teamworking: a) -0.346*; b) -0.183; c) -0.369* 3) Appraisal: a) -0.391*; b) -0.372*; c) -0.340 * | Not presented | There is a significant association between sophistication of training policy and mortality index | 3 | 3 | 0 | All positive |
| West et al (2006) | Cross-sectional, 81 facilities, HIC | England | Public | Hospitals | 1) Standardised mortality ratio: 0.078** | Not presented | The HR system accounts for 7.8% proportion of the variance in subsequent mortality rates | 1 | 1 | 0 | All positive |
| Yoo et al (2019) | Cross-sectional, 20 facilities, LMIC | India | Public and private | Hospitals | Odds of 30-day Major Adverse Cardiovascular Events in tercile 3: 1.02 | 95% CI: 0.34-3.12 | Moving from the first to third tercile of management score is not associated with a significant difference in odds of 30-day Major Adverse Cardiovascular Events | 1 | 0 | 0 | All null |
| Zhu et al (2021) | Cross-sectional, 95 facilities, LMIC | China | Public | Hospitals | 1) In-hospital death rate (%): -1.1 2) Nosocomial infection rate (%): 0.3 | 95% CI:  1) -3.5-1.4  2) -3.2-3.9 | No evidence of an association between management score and in-hospital death rate | 2 | 0 | 0 | All null |

#### Figure A1 Management-quality association, by type of survey method
